# Classification of tumor from computed tomography images: A brain-inspired multisource transfer learning under probability distribution adaptation

**DOI:** 10.3389/fnhum.2022.1040536

**Published:** 2022-10-20

**Authors:** Yu Liu, Enming Cui

**Affiliations:** ^1^School of Electronic Information and Automation, Guilin University of Aerospace Technology, Guilin, Guangxi, China; ^2^Department of Radiology, Jiangmen Central Hospital, Jiangmen, Guangdong, China

**Keywords:** gastric tumor classification, multisource transfer learning, distribution adaptation, extreme learning machine, brain-inspired analysis

## Abstract

Preoperative diagnosis of gastric cancer and primary gastric lymphoma is challenging and has important clinical significance. Inspired by the inductive reasoning learning of the human brain, transfer learning can improve diagnosis performance of target task by utilizing the knowledge learned from the other domains (source domain). However, most studies focus on single-source transfer learning and may lead to model performance degradation when a large domain shift exists between the single-source domain and target domain. By simulating the multi-modal information learning and transfer mechanism of human brain, this study designed a multisource transfer learning feature extraction and classification framework, which can enhance the prediction performance of the target model by using multisource medical data (domain). First, this manuscript designs a feature extraction network that takes the maximum mean difference based on the Wasserstein distance as an adaptive measure of probability distribution and extracts the domain-specific invariant representations between source and target domain data. Then, aiming at the random generation of parameters bringing uncertainties to prediction accuracy and generalization ability of extreme learning machine network, the 1-norm regularization is used to implement sparse constraints of the output weight matrix and improve the robustness of the model. Finally, some experiments are carried out on the data of two medical centers. The experimental results show that the area under curves (AUCs) of the method are 0.958 and 0.929 in the two validation cohorts, respectively. The method in this manuscript can provide doctors with a better diagnostic reference, which has certain practical significance.

## Introduction

Gastric cancer (GC) and primary gastric lymphoma (PGL) are the two most common malignant gastric tumors. The clinical manifestations of these two tumors are very similar (Sun et al., 2021), while their treatment strategies are different. Surgical resection remains the main treatment option for GC, especially for patients who may be cured by radical resection. However, the best PGL treatment options are chemotherapy or radiotherapy. In addition, PGL lesions are generally located in the submucosa of the gastric wall, which makes biopsy testing unable to accurately locate lesions and leads to high false-negative rates (Jung et al., 2016). Noninvasive computed tomography (CT), widely used for differential and preoperative diagnoses, therapeutic evaluation, and staging in patients with gastric malignancies, can help find tumor lesions (Tsurumaru et al., 2016). However, distinguishing GC and PGL by CT signs of lesion distribution, irregular gastric wall thickness and enhancement pattern is difficult (Park et al., 2010). Thus, considering the differences in clinical management between PGL and GC, it is of great value to differentiate PGL and GC preoperatively, which may facilitate clinical decision-making.

Brain-like intelligent decision-making is a prevailing trend in today’s world. Inspired by bionics and computer science, the deep neural network has become one of the main means to realize human-like decision-making and control, and is a popular computer-aided diagnosis technology in medical imaging diagnosis. Due to its excellent feature learning ability, deep neural network has been continuously applied in the classification and preoperative diagnosis of diseases (Tian et al., 2019; Feng et al., 2020a,b; Kosaraju et al., 2020). However, the training dataset size is crucial to building a robust model, while obtaining large numbers of medical images is difficult in clinical practice. Thus, developing a method to improve the deep learning model performance is necessary. To improve the model’s performance under small samples of medical datasets, transfer learning technology has been widely used.

Transfer learning, which are inspired by the inductive reasoning learning of the human brain, improves model performance in target tasks by transferring features from source tasks that have already been learned. Moreover, transfer learning has been gradually applied in recent years to many medical image analytical fields (e.g., image segmentation, lesion localization, and lesion pattern recognition) (Van Opbroek et al., 2015; Pan and Yang, 2020). Here, the paradigm of fine-tuning parameters on the target data after pretraining based on the ImageNet dataset is the most common (Romero et al., 2020; Song et al., 2021). However, studies have shown that the distribution similarity of the source and target domains is a key factor in determining the effect of transfer (Kornblith et al., 2018; Raghu et al., 2019). When there exists a large domain shift between the source and target domains, transfer learning based on the pretraining and fine-tuning paradigm may cause negative transfer (Raghu et al., 2019). In addition, based on a single-source domain, the model may learn the basic texture and color features of natural images so that the discriminant ability of the model tends to only single-source domain representation (Li and Wang, 2022), and the generalization performance may be poor.

Generally, data from multisource domains with different data distributions can be collected in practical application scenarios. The knowledge and internal relationship learned from multisource domains can be better used to assist the target task (Fang et al., 2021). Therefore, transfer learning methods with multisource domains should have more potential for the prediction performance improvement of target tasks. Due to this advantage and potential, multisource transfer learning has gradually attracted the attention of researchers and has been widely used in some classification tasks (Li et al., 2019b; Zhang et al., 2021). However, for multisource transfer learning, it is necessary to find a method for learning a discriminative model in the presence of a domain shift between the multisource and target domains to better make full use of multiple source domains. For example, in Fang et al. (2021), a multisource ensemble transfer learning framework (MultiLSTM-DANN) was proposed, which measures the marginal probability distribution between different domains by the maximum mean discrepancy (MMD) and transfers the features with less distribution difference to build a classification model. In addition, some studies (Li et al., 2019a; Wang et al., 2019) utilize joint probability adaptation (JDA) to further analyze the marginal and conditional probability distributions of the features to reduce the distribution differences between the source and target domains. The above researches attempt to map all source and target domain data into a common feature space to reduce domain distribution shifts and learn common domain-invariant representations. However, it is not easy to learn domain-invariant representations, even for one single-source and one target’s domain data (Zhu et al., 2019). Moreover, only considering the single distribution of data makes it difficult to meet the actual situation, and we should combine different data characteristics to analyze the data distribution. Hence, we attempt to use the adaptive distribution adaptation method to analyze the difference between each pair of source and target domains and extract domain-invariant representations.

Motivated by the above problems, we propose an extreme learning machine based on adaptation multiple spaces feature and L1-norm regularization (AMSF-L1ELM), and the contributions of this manuscript are summarized as follows:

(1) *Adaptation multisource transfer learning feature extraction network*. By simulating the multi-modal information learning and transfer mechanism of human brain, we propose a feature extraction network for gastric tumor CT images that adaptively considers the difference between the marginal distribution (related to the data generation mechanism) and conditional distribution (related to specific downstream tasks) of multisource data. ➀ The network takes the maximum mean difference based on the Wasserstein distance to adaptively evaluate the probability distribution of each pair of source and target domains and extracts the domain-specific invariant representations between each pair of source and target domains. ② To reduce the misclassification of target samples near domain-specific decision boundaries, this study adopts an ensemble learning classifier for the comprehensive evaluation of target samples. Based on the network, deep learning features fused with multisource domain information are extracted to pave the way for classification tasks.
(2) *Extreme learning machine based on L1-norm regularization*. To achieve the effective classification of deep features, this study uses a lightweight neural network extreme learning machine to build a classification model. Aiming at the problems that the output of an extreme learning machine is prone to random fluctuation and poor generalization performance, L1-norm regularization is used to implement sparse constraints of the output weight matrix and improve the model robustness.

## Materials and methods

**FIGURE 1:**
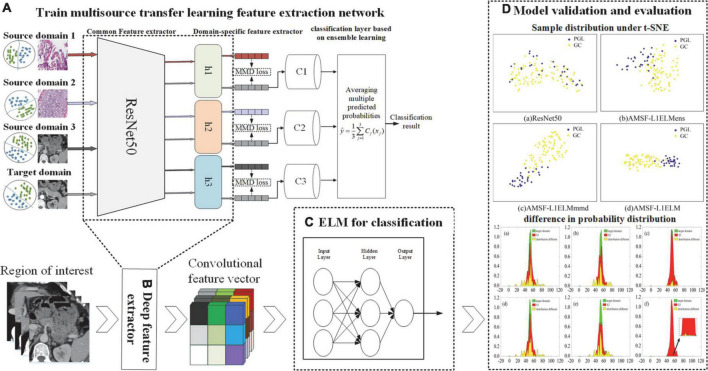
The overall structure of adaptation multiple spaces feature and L1-norm regularization (AMSF-L1ELM). **(A)** Multisource transfer feature extraction network framework. **(B)** Deep feature extractor. **(C)** Extreme learning machine for classification. **(D)** Model validation and evaluation.

The AMSF-L1ELM includes three parts: (1) training the multisource transfer learning feature extraction network based on feature distribution dynamic alignment, and extracting the deep learning features, (2) feature classification algorithm based on extreme learning machine with L1-norm regularization, and (3) model validation and evaluation. The overall structure of AMSF-L1ELM is shown in Figure 1.

### Multisource transfer learning feature extraction network

**FIGURE 2:**
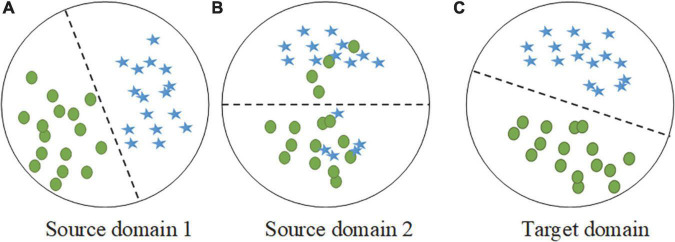
Examples of two different source domains and the target domain during distribution adaptation. **(A)** The sample distribution of source domain 1. **(B)** The sample distribution of source domain 2. **(C)** The sample distribution of target domain.

Transfer learning has been widely studied for many years, and its effectiveness has been verified by researchers. The performance improvement of multisource transfer learning largely depends on the data distribution between the source and target domains, and some studies have shown that the distribution similarity of the source and target domains is a key factor in determining the effect of transfer (Kornblith et al., 2018; Raghu et al., 2019). It is worth noting that the distributions of the source and target domains are different in practical application scenarios and may satisfy different probability distributions. As shown in Figure 2, when the overall distributions of source domain 1 and the target domain are similar, the local conditional probability distribution should be focused on. In contrast, the overall distributions of source domain 2 and the target domain are different, and the marginal distribution should be prioritized. Thus, the key to successful transfer is to adaptively measure and reduce the distribution divergence between the source and target domains.

**FIGURE 3:**
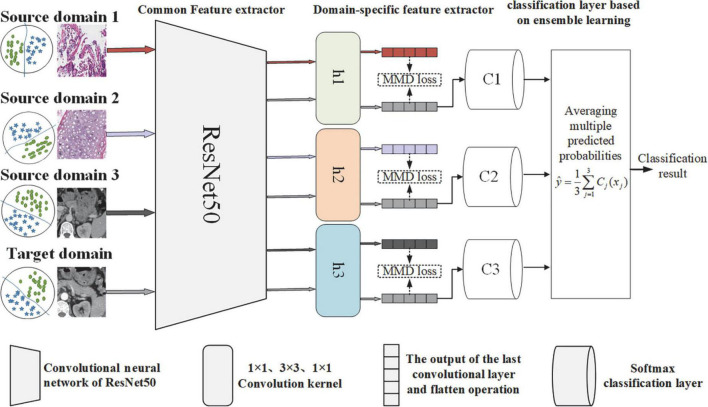
Multisource transfer feature extraction network framework.

Considering clinical practice, doctors often need to use a variety of means for disease diagnosis, such as lesion screening based on multisequence CT images (such as arterial phase and venous phase) and then pathological diagnosis based on WSI images. Additionally, the effectiveness of the transfer learning model, which was based on single-source domain WSI of gastric tissue and WSI of lung tissue, was preliminarily validated according to our previous work (Feng et al., 2021). Considering the above observations, this manuscript plans to select the gastric WSIs (source domain 1), lung WSIs (source domain 2) and arterial phase CT images of gastric cancer (source domain 3) as the source domain data, and proposes a multisource transfer learning feature extraction network, as shown in Figure 3. The goal of the feature extraction network framework is to dynamically measure the marginal and conditional distributions between the source and target domains to adaptively minimize the distribution difference and extract more effective deep learning features.

#### Domain feature distribution alignment

##### Common feature extractor

The residual network (ResNet) (He et al., 2016) avoids network degradation with the introduction of a skip connection structure and has the advantage of optimizing the fitting ability. Considering the computer equipment and prediction task requirements, we use ResNet50 as the common feature extraction subnetwork, which maps the images from the original feature space into a common feature space. To prevent overfitting the model, the parameters of the network are initialized based on the model pretrained by the ImageNet dataset.

##### Domain-specific feature extractor

Considering domain-invariant representations for each pair of source and target domains is easier than extracting common domain-invariant representations for all domains. Thus, we map each pair of source and target domains into a specific feature space for analysis. In a specific feature space, we aim to adaptively eliminate distribution differences between source and target domains and extract domain-invariant representations. MMD is often used to construct two-sample tests and determine whether the two data distributions are the same (Zhao et al., 2021). Here, the MMD is used as the basic measure of the feature distribution in the domain-specific feature space, and we propose an adaptive MMD feature distribution alignment algorithm based on the Wasserstein distance.

Maximum mean discrepancy is a kernel two-sample test that rejects or accepts the null hypothesis p = q based on the observed samples. The basic idea is that if the generating distributions are identical, all the statistics are the same. Formally, MMD defines the following difference measure:


(1) $$M_{H}\left(p\,\left(X^{\text{s}}\right),q\,\left(X^{\text{t}}\right)\right)={||\frac{1}{n}\,\sum_{i=1}^{n}\phi \,\left(x_{i}^{\text{s}}\right)-\frac{1}{m}\,\sum_{j=1}^{m}\phi \,\left(x_{j}^{\text{t}}\right)||}_{\text{H}}^{2}$$

where $X^{\text{s}}={\left\{x_{i}^{\text{s}}\right\}}_{i=1}^{\left|X^{\text{s}}\right|}$ is a set of source domains with probability distribution *p* and $X^{\text{t}}={\left\{x_{j}^{\text{t}}\right\}}_{j=1}^{\left|X^{\text{t}}\right|}$ is target domain with probability distribution *q*. H denoting the reproducing kernel Hilbert space (RKHS) with a characteristic kernel *k*,*n* = |*X*^s^| is the number of samples in the source domain, *m* = |*X*^t^| is the number of samples for the target domain. ϕ(●) is the mapping function from the original space to the RKHS, and satisfies the following relation: < ϕ(*x*),ϕ(*y*) >_*H*_ = *k*(*x*, *y*). *k*(*x*, *y*) is a Gaussian kernel function, namely:


(2) $$k\,\left(x,y\right)=\exp\left(-{||x-y||}^{2}\,/\,2\,\sigma^{2}\right)$$

where σ represents the size of the Gaussian kernel. Combining Equations (1) and (2), the MMD between each source and target domain is defined as:


(3) $$\begin{array}{c} M_{H}\,\left(p\,\left(X^{\text{s}}\right),q\,\left(X^{\text{t}}\right)\right)=\frac{1}{n^{2}}\,\sum_{i=1}^{n}\sum_{j=1}^{n}k\,\left(x_{i}^{\text{s}},x_{j}^{\text{s}}\right)\\ -\frac{1}{m\,n}\,\sum_{i=1}^{n}\sum_{j=1}^{m}k\,\left(x_{i}^{\text{s}},x_{j}^{\text{t}}\right)\\ +\frac{1}{m^{2}}\,\sum_{i=1}^{m}\sum_{j=1}^{m}k\,\left(x_{i}^{\text{t}},x_{j}^{\text{t}}\right) \end{array}$$

In clinical practice, the distribution of multisource medical data may be different. For example, since CT and WSI are generated by different mechanisms, their marginal probability distributions may be different. In contrast, venous phase images and arterial phase images were obtained from the same patient at different scan times, and their local conditional probability distributions may be more different. Based on the above assumptions, this study takes MMD as the basic data distribution measurement tool and introduces the probability distribution adaptation factor μ to adaptively measure the marginal and conditional distributions of the data, defined as follows:


(4) $$\begin{array}{c} M_{\text{MMD}}\left(X^{s},X^{t}\right)=\left(1-\mu \right)M_{\text{H}}\left(p\left(X^{\text{s}}\right),q\left(X^{\text{t}}\right)\right))\\ +\mu \,\sum_{l=0}^{1}M_{H}^{\left(l\right)}\,\left(p\,\left(Y^{\text{s}\,\left(l\right)}|X^{\text{s}\,\left(l\right)}\right),q\,\left(Y^{\text{t}\,\left(l\right)}|X^{\text{t}\,\left(l\right)}\right)\right) \end{array}$$

where μ ∈ [0,1] is the probability distribution adaptation factor, and *l* ∈ {0,1} denotes the sample class. *p*(*X*^s^) and *q*(*X*^t^) represent the marginal distribution of source and target domain data, and *p*(*Y*^s^|*X*^s^) and *q*(*Y*^t^|*X*^t^) represent the conditional distribution of source and target domain data.

When μ→0, the difference in the global distribution between the source and target domains is large, and the marginal distribution adaptation is more important. When μ→1, it indicates that the difference in the local distribution between the source and target domain data is higher, and the conditional distribution is more important. When μ→0.5, the marginal and conditional distributions of the representation data are equally important, and JDA studies this work. Inspired by but different from JDA, MMD is used as the basic measure of feature marginal and conditional distribution, and then Wasserstein distance (Lichtenegger and Niedzialomski, 2019) is used to calculate the probability adaptation factor μ for weighing the importance of conditional and marginal distribution in this study.

The Wasserstein distance is based on the optimal transport theory and aims to adapt the difference between the data probability distributions with minimum cost. It is defined as follows:


(5) $$W\,\left(X^{s},X^{t}\right)=\underset{\nu ∼\prod \left(p,q\right)}{\inf}{\text{E}}_{\left(x,y\right)∼\nu }\,\left[||x-y||\right]$$

where ∏(*p*,*q*) is the set of all possible joint distributions combined by *p* and *q* distributions. For each possible joint distribution ν, we can obtain a sample *x* and *y* from it and calculate the distance between the two samples ||*x*−*y*||. Then, we can calculate the expected value E_(*x*,*y*)∼ν_[||*x*−*y*||] of the sample under the distribution ν. In all possible distributions, the lower bound that can be taken for this expected value is the Wasserstein distance.

Based on Eq. (5), we calculate the global Wasserstein distance between the source and target domains as the weight of the marginal probability, which is written as *W*_*g*_. Inspired by but different from Wang (Wang et al., 2017), the Wasserstein distance of the *l*−*th* class from source domain *X^s^* and target domain *X^t^* is the weight of the conditional probability distribution, which is written as *W*_*l*_ = *W*(*X*^s(l)^,*X*^t(l))^. Further calculation of the probability adaptation factor μ is shown in the following formula:


(6) $$\mu =\frac{\sum_{l=0}^{1}W_{l}}{W_{g}+\sum_{l=0}^{1}W_{l}}$$

Combining Eqs (4) and (6), the final difference in the probability distribution between the source and target domain data is obtained as follows:


(7) $$M_{\text{MMD}}=\frac{W_{g}}{W_{g}+\sum_{l=0}^{1}W_{l}}\,M_{\text{H}}+\frac{\sum_{l=0}^{1}W_{l}}{W_{g}+\sum_{l=0}^{1}W_{l}}\,\sum_{l=0}^{1}M_{\text{H}}^{\left(l\right)}$$

Finally, we use Eq. (7) as the estimate of the discrepancy between each pair of source and target domains. The MMD loss for three pairs of source and target domains is reformulated as:


(8) $$l_{M\,M\,D}=\frac{1}{3}\,\sum_{i=1}^{3}{\left\{M_{\text{MMD}}^{\left(i\right)}\right\}}_{i}$$

#### Deep learning feature extraction

For the domain-specific feature extractors, three groups of target domain features are generated. To reduce the misclassification of target samples near the domain-specific decision boundary, this study adopts the idea of ensemble learning, and we train three subpredictors ${\left\{C_{j}\right\}}_{j=1}^{3}$ based on three group features of the target domain. Each predictor *C*_*j*_ is a softmax classifier and receives the target domain-invariance feature from the *j*−*th* domain-specific feature extractors. Then, the features are input into multiple trained classifiers for prediction, and the average of the predicted results for three classifiers is the final prediction, as follows:


(9) $$\hat{y}=\frac{1}{3}\,\sum_{j=1}^{3}C_{j}\,\left(x_{j}\right)$$

where *x*_*j*_ are target domain-invariance features from the*j*−*th*domain-specific feature extractor. After obtaining the prediction$\hat{y}$ and the corresponding truth label *y*, we have the experience loss:


(10) $$l_{\text{task}}=J\,\left(\hat{y},y\right)$$

where*J*(*)represents the cross-entropy loss function.

Based on Eqs (8–10), the total loss function of the feature extraction network can be obtained as follows:


(11) $$l_{\text{total}}=\alpha \,l_{\text{MMD}}+l_{\text{task}}$$

where α is the hyperparameter that controls the impact of MMD loss.

**FIGURE 4:**
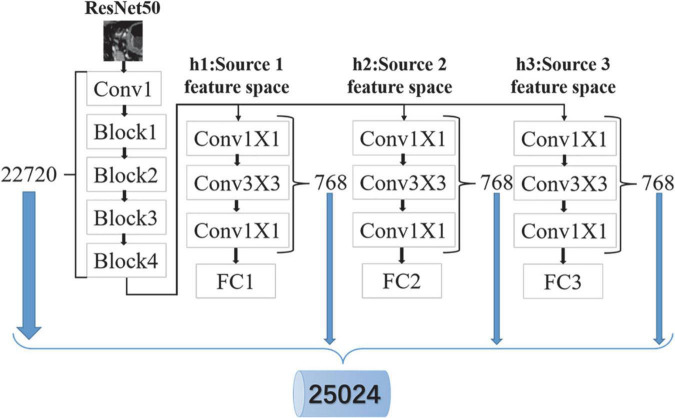
Deep learning feature extraction.

By minimizing Eq. (11), the feature extraction network is trained (see section “Experimental parameters” for specific training details). In this manuscript, the convolution kernel in the feature extraction network is used as a feature extractor to extract specific features. As shown in Figure 4, there are 21,440 features in total, including 19,136 from the common feature extraction network and 2,304 from three domain-specific feature extraction subnetworks. To reduce the feature redundancy and improve the running speed of the classification model, the maximum relevance and minimum redundancy (mRMR) algorithm (Bugata and Drotar, 2020) is used to select the top 10% features with higher relevance to the label for classification.

### Extreme learning machine based on 1-norm regularization

An extreme learning machine (ELM), which is an efficient feedforward neural network with a single hidden layer, has the characteristics of high learning speed and strong global search ability (Shi et al., 2018). For*M*training samples ${\left\{\left(x_{i},y_{i}\right)\right\}}_{i=1}^{M}$, *x*_*i*_ = [*x*_*i*1_,…, *x*_*in*_]^T^ ∈ *R^n^* is the training sample feature vector, and the label corresponding training sample *x*_*i*_ is *y*_*i*_ = [*y*_*i*1_,…, *y*_*im*_]^T^ ∈ *R^m^*. The expression of the ELM output with*L*hidden layer neurons is:


(12) $$f\,\left(x\right)=\sum_{i=1}^{L}\varphi_{i}\,g\,\left(\omega_{i}●x_{i}+b_{i}\right)=y_{i},x_{i}\in R^{n},\omega_{i}\in R^{n},\varphi_{i}\in R^{m}$$

where ω_*i*_ is the weight vector of the input layer neurons, and *b*_*i*_ is the bias of *i*−*th* hidden layer neurons. φ_*i*_ is the output weight vector, and ω_*i*_●*x*_*j*_ represents the inner product of ω_*i*_ and *x*_*j*_. *g*(●) is the activation function of the hidden layer.

Equation (12) is simplified and can be expressed as Eq. (13):


(13) G⁢φ=Y

where *G* represents the output matrix of the hidden layer, φ represents the weight matrix, and *Y* represents the expected output matrix:


(14) $$G={\left[\begin{array}{ccc} g_{i}\,\left(\omega_{1}●x_{1}+b_{1}\right) & \cdots & g_{i}\,\left(\omega_{K}●x_{1}+b_{L}\right)\\ \vdots & \ddots & \vdots \\ g_{i}\,\left(\omega_{1}●x_{N}+b_{1}\right) & \cdots & g_{i}\,\left(\omega_{K}●x_{N}+b_{L}\right) \end{array}\right]}_{N\times L}$$

(15) $$\varphi =\left[\begin{array}{c} \varphi_{1}^{\text{T}}\\ \vdots \\ \varphi_{K}^{\text{T}} \end{array}\right],Y=\left[\begin{array}{c} y_{1}^{\text{T}}\\ \vdots \\ y_{L}^{\text{T}} \end{array}\right]$$

When *g*(●) is infinitely differentiable, the ELM model training process can be approximated as solving the least squares solution of linear *G*φ = *Y* with random parameters ω_*i*_ and *b*_*i*_, as shown in Eq. (16):


(16) $$\hat{\varphi }=G^{†}\,Y$$

where *G*^†^ is the generalized inverse of the Moore-Penrose pseudoinverse for the hidden layer output matrix *G*.

The traditional ELM theory is based on empirical risk minimization theory, which have three basic steps: A random projection of the input space followed by some nonlinear operation and finally a linear output layer of weights. The basic ELM uses pseudo matrix inverse to estimate the output layer weights which usually leads to over fitting and has poor stability. To improve the model performance, structural risk minimization theory (Hao et al., 2022) is adopted. Our perspective to the output layer weight estimation in ELM is approached from the feature selection point of view. The random layer followed by the nonlinear activations maps the feature space to another high dimensional linearly separable space. Looking at the outputs of the hidden neurons as the input features in this new space, and since many of the neurons produce noisy outputs, the problem can be formulated as a linear feature selection one. In the sense that, we would like to find the minimum number of features (hidden neurons) such that the linear classifier performance is optimized. Therefore, L1-norm regularization is used to implement sparse constraints of the output weight matrix to improve the performance of the model. The specific expression is as follows:


(17) $$\min_{\varphi }||G\,\varphi -Y||+\beta \,{||\varphi ||}_{1}$$

Solve the above formula further:


(18) $$\hat{\varphi }={\left(G^{\text{T}}\,\text{G}+\frac{1}{\beta }\,\text{I}\right)}^{-1}\,{\text{G}}^{\text{T}}\,\text{Y}$$

where β are the regularization parameters and *I* is the identity matrix.

When the number of hidden layer nodes, regularization parameters and activation function are set, ω_*i*_ and *b*_*i*_ are randomly generated. Then, the output weight is solved based on Eq. (18), and the predicted value of the model is calculated.

## Experiments and evaluations

### Data preparation

#### Source domain datasets

Source domain 1 (**S1**) is gastric cancer WSIs from the public dataset of the MARS data science platform (45,797 images of GC and 12,340 normal images). Source domain 2 (**S2**) is lung WSIs from The Cancer Genome Atlas (TCGA) (290,558 lung adenocarcinoma images, 285,995 lung squamous cell carcinoma images), and source domain 3 (**S3**) is gastric artery phase CT images from Jiangmen Central Hospital (including 2,465 images of GC, 1,262 images of PGL).

#### Target domain datasets

The target domain data (**T**) for this study are gastric venous phase CT images, which were collected from two medical centers and include 184 patients from center 1 and 95 patients from center 2. There were 110 patients in the training set (1,301 images of GC, 604 images of PGL), 74 patients in the internal validation cohort (1,280 images of GC, 491 images of PGL) and 95 patients in the external validation cohort (4,976 images of GC, 2,677 images of PGL).

Table 1 shows the dataset information.

**TABLE 1:** Basic information about multisource datasets.

Domain	Domain name	Domain description	Data distribution	
Source domain	S1	Gastric WSIs	45,797 images of GC	12,340 normal images
	S2	Lung WSIs	290,558 lung adenocarcinoma images	285,995 lung squamous cell carcinoma images
	S3	Gastric artery phase CT images	2,465 images of GC	1,262 images of PGL
Target domain	T: Center1	Gastric venous phase CT image	2,581 images of GC	1,095 images of PGL
	T: Center2	Gastric venous phase CT image	4,976 images of GC	2,677 images of PGL

### Experimental parameters

The samples of the target domain were collected by Toshiba Aquilion One 64-slice spiral CT. The scanning parameters were as follows: tube voltage 120 kV, tube current automatic, detector collimation 64 mm × 0.625 mm or 192 mm × 0.625 mm, field of view 350 mm × 350 mm, pitch 0.656 or 0.7, matrix 512 × 512, slice spacing 3 mm, slice thickness 3 mm, and reconstruction slice thickness 3 mm. A plain scan was performed first, and then a contrast medium (1.5 ml/kg, Ultravist, Bayer Schering) was injected through the antecubital vein at a rate of 3.0–3.5 ml/s with a high-pressure syringe. The arterial phase and venous phase images were scanned at 30 and 60 s, respectively.

The training parameters of the multisource transfer learning feature extraction network are as follows: the common feature extractor is obtained by fine-tuning the ImageNet pretraining network parameters based on ResNet50. A domain-specific feature extraction subnetwork, including 1 × 1, 3 × 3, and 1 × 1 convolution layers, is proposed to extract domain-specific features. The whole network chooses the stochastic gradient descent (SGD) algorithm with momentum as the optimizer (momentum is 0.9, weight attenuation is 10^–4^, the initial learning rate of the common feature extraction network is 0.001, and the initial learning rate of the domain-specific feature extraction subnetwork is 0.01) to train, and the loss function is cross entropy. The batch size is set to 32, and the training rounds are 2,000. To suppress the image noise at the initial stage, the regularization parameter α is selected based on the following rules (Ganin and Lempitsky, 2015). $\alpha =\frac{2}{\exp\left(-\theta \times i\,t\,e\,r\right)}-1$, where θ = 10, and *iter* represents the number of iterations. In this manuscript, the PyTorch framework is used to implement the proposed method, and the training is performed on an RTX A6000 graphics card. In addition, After many experiments, the regularization parameter β = 0.05of ELM.

### Result evaluation index

To evaluate the performance of the diagnosis model, the sensitivity (Sen), specificity (Spy), accuracy (Acc), precision (Pre) and F1 value are measured (Bradley, 1997), and the calculation equations are as follows:


(19) $$\left\{ \begin{array}{c} S\,e\,n=\frac{T\,P}{T\,P+F\,N}\\ S\,p\,e=\frac{T\,N}{T\,N+F\,P}\\ A\,c\,c=\frac{T\,P+T\,N}{T\,P+F\,N+T\,N+F\,P}\\ \mathrm{Pre}=\frac{T\,P}{T\,P+F\,P}\\ F\,1=2\times \frac{\mathrm{Pre}\times S\,e\,n}{\mathrm{Pre}+S\,e\,n} \end{array}\right.$$

where TP is the number of samples correctly classified as GC, TN is the number of samples correctly classified as PGL, FP is the number of samples misclassified as GC, and FN is the number of samples misclassified classified as PGL.

In addition, the algorithm performance is evaluated by the receiver operating characteristic curve (ROC) (Bradley, 1997), and the area under curve (AUC) is usually used to quantify the effect of the algorithm. The larger the AUC (0 ≤ AUC ≤ 1), the better the classification performance.

## Results and analysis

### Comparison with state-of-the-art methods

To verify the superiority of the proposed AMSF-L1ELM, several state-of-the-art methods are used for comparison in two validation cohorts. Representative methods include the classic radiomics signature (RS) (Feng et al., 2021) based on radiomics features, a clinical model (CM) based on subjective signs and clinical information (Feng et al., 2021), Yu’s et al. (2019) transfer learning with a dynamic adversarial adaptation network (DAAN), Cui’s et al. (2020) batch nuclear-norm maximization (BNM), and Zhu’s et al. (2021) deep subdomain adaptation network (DSAN).

From these results in Table 2, we can obtain the following insightful observations.

**TABLE 2:** Results of comparison with state-of-the-art methods.

Dataset	Models	AUC	Sen	Spe	Acc	Pre	F1
Internal validation cohort	CM	0.820	0.836	0.680	0.784	0.894	0.864
	RS	0.837	0.735	**0.962**	0.813	0.824	0.777
	DAAN	0.859	0.783	0.770	0.778	0.853	0.816
	BNM	0.840	0.739	0.816	0.767	0.873	0.800
	DSAN	0.783	0.844	0.557	0.738	0.765	0.803
	AMSF-L1ELM	**0.958**	**0.980**	0.920	**0.960**	**0.941**	**0.960**
External validation cohort	CM	0.717	**0.942**	0.512	0.747	0.890	0.915
	RS	0.814	0.596	**0.907**	0.737	0.959	0.735
	DAAN	0.822	0.772	0.664	0.706	0.588	0.667
	BNM	0.789	0.802	0.596	0.675	0.551	0.653
	DSAN	0.789	0.852	0.568	0.677	0.550	0.668
	AMSF-L1ELM	**0.929**	0.885	0.814	**0.853**	**0.973**	**0.927**

The significance of bold values indicates the maximum value of the indicator in the internal and external validation cohort, respectively.

(1) Compared with RS, AMSF-L1ELM is based on a deep learning framework, and specific features related to tumors can be extracted in higher dimensions. The CM, which is based on the patient’s clinical characteristics and CT signs, is highly dependent on the experience of doctors.
(2) Dynamic adversarial adaptation network and BNM learn a global domain shift, i.e., align the global source and target distributions without considering the relationships between two subdomains within the same category of different domains, leading to unsatisfactory transfer learning performance without capturing fine-grained information.
(3) Compared with the proposed AMSF-L1ELM, the DSAN learns a transfer network by aligning the relevant subdomain distributions based on a local maximum mean discrepancy, and it matches distributions without considering domain-specific decision boundaries between classes.
(4) The proposed AMSF-L1ELM method obtains the best prediction results on the GC and PGL classification tasks, verifying its effectiveness and superiority.

### Ablation experiment

To verify the effectiveness of the algorithm, ablation experiments are carried out for the following model: (1) ResNet50, the baseline model; (2) AMSF, the end-to-end multisource transfer learning feature classification model; (3) AMSF-L1ELM_*ens*_, without considering the MMD loss; (4) AMSF-L1ELM_*mmd*_, without considering the ensemble learning classifier; (5) AMSF-ELM, without considering the L1-norm regularization; and (6) AMSF-L1ELM, the method of this manuscript.

Based on the same parameters and datasets, the above models are compared and analyzed (Table 3). With the introduction of probability distribution adaptation and ensemble learning classifiers, the results of the proposed method (AMSF-L1ELM) are improved to different degrees. The results showed that the dynamic adaptation of the marginal probability distribution and the conditional probability distribution has a certain effect, and the ensemble learning classifier focuses on punishing the misjudgment of the class boundary samples. In addition, compared with the AMSF-ELM model, AMSF-L1ELM with L1-norm regularization improves the AUCs of the classifier by 0.138 and 0.116 in the internal and external validation cohorts, respectively.

**TABLE 3:** Diagnostic performance of different structural models.

Dataset	Models	AUC	Sen	Spe	Acc	Pre	F1
Internal validation cohort	ResNet50	0.816	0.837	0.760	0.811	0.654	0.734
	AMSF	0.917	0.833	0.838	0.835	0.898	0.864
	AMSF-L1ELM_*ens*_	0.898	0.878	0.800	0.851	0.896	0.887
	AMSF-L1ELM_*mmd*_	0.929	0.959	0.840	0.919	0.922	0.940
	AMSF-ELM	0.820	0.878	0.720	0.824	0.856	0.867
	AMSF-L1ELM	**0.958**	**0.980**	**0.920**	**0.960**	0.960	**0.970**
External validation cohort	ResNet50	0.780	0.731	0.721	0.726	0.955	0.828
	AMSF	0.756	0.585	0.739	0.661	0.700	0.637
	AMSF-L1ELM_*ens*_	0.869	0.865	0.791	0.832	0.834	0.849
	AMSF-L1ELM_*mmd*_	0.902	0.750	**0.907**	0.821	0.907	0.821
	AMSF-ELM	0.813	0.808	0.744	0.779	0.792	0.800
	AMSF-L1ELM	**0.929**	**0.885**	0.814	**0.853**	0.852	**0.868**

The significance of bold values indicates the maximum value of the indicator in the internal and external validation cohort, respectively.

### Effect of different source domains on the algorithm

Studies have shown that when the source domain data are more similar to the target domain data, the effect of transfer learning is better. This study analyzed the impact of different source domains, and the comparison models are shown in Table 4. This manuscript analyzes the single-source domain, double-source domain and three-source domain.

**TABLE 4:** Effect of different source domains on the algorithm.

Dataset	Models	AUC (95% CI)	Sen	Spe	Acc	Pre	F1
Internal validation cohort	S1 → T	0.824	0.959	0.600	0.838	0.890	0.923
	S2 → T	0.822	0.694	0.840	0.743	0.896	0.782
	S3 → T	0.857	0.978	0.720	0.892	0.811	0.887
	S1, S2 → T	0.921	0.980	0.880	0.946	0.941	0.960
	S1, S3 → T	0.931	0.857	0.920	0.878	0.954	0.903
	S2, S3 → T	0.863	0.816	0.800	0.811	0.889	0.851
	S1, S2, S3 → T	**0.958**	**0.980**	**0.920**	**0.960**	**0.960**	**0.970**
External validation cohort	S1 → T	0.818	0.808	0.744	0.779	0.810	0.809
	S2 → T	0.805	0.731	0.814	0.768	0.827	0.776
	S3 → T	0.841	0.692	**0.977**	0.821	0.948	0.800
	S1, S2 → T	0.890	0.808	0.884	0.842	0.895	0.849
	S1, S3 → T	0.900	0.846	0.861	0.853	0.881	0.863
	S2, S3 → T	0.856	0.885	0.698	0.800	0.780	0.829
	S1, S2, S3 → T	**0.929**	**0.885**	0.814	**0.853**	0.852	**0.868**

The significance of bold values indicates the maximum value of the indicator in the internal and external validation cohort, respectively.

For the single-source domain, the results show that the model based on S3 is superior to the prediction results based on S1 and S2, and the AUCs in the two validation cohorts were 0.857 and 0.841, respectively. The main reason is that S3 and T are homologous images, and the overall distribution between S3 and T is more similar. The comparative analysis of a single-source domain proves again that the similarity between the source and target domains contributes to the improvement of the transfer effect. Furthermore, this study analyzes the diagnostic performance of the model under the double-source domain. S1, which reflects the gastric tumor information of local lesions from a micro level, may be relevant to the task. The results show that the model (S1, S3 → T) fuses the information of arterial phase CT and WSI of GC, and it achieves good prediction performance in both validation cohorts (AUC = 0.931 and 0.900).

Notably, although S2 is the WSI of the lung and is a different organ than the stomach, S2 and T are both medical images, and they may contain some basic medical features. Thus, with the introduction of S2, the AUCs of AMSF-L1ELM (S1, S2, S3 → T) in the two validation cohorts were best (0.958 and 0.929, respectively).

### Visualization analysis of parameters and features

**FIGURE 5:**
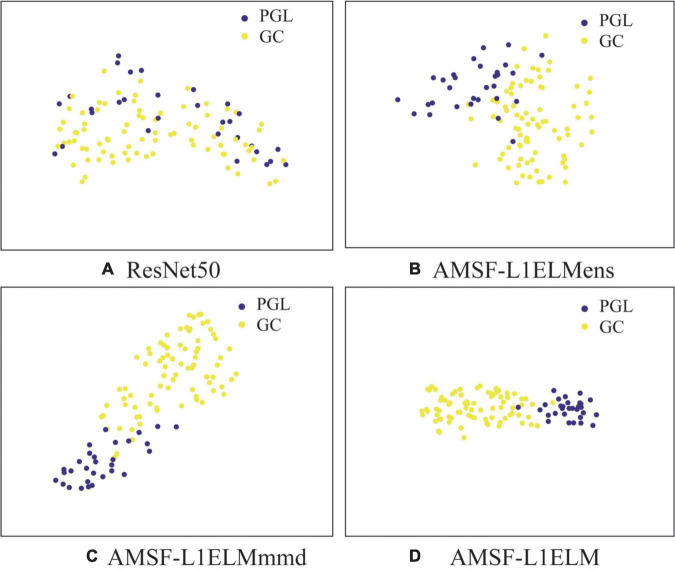
Sample distribution under different model features. **(A)** The baseline model ResNet50. **(B)** The model without considering the maximum mean discrepancy (MMD) loss. **(C)** The model without considering the ensemble learning classifier. **(D)** The method of this manuscript. GC, gastric cancer; PGL, primary gastric lymphoma.

In Figure 5, we visualize the latent representations for ResNet50, AMSF-L1ELM_*ens*_, AMSF-L1ELM_*mmd*_, and AMSF-L1ELM by using the t-SNE algorithm (Liu et al., 2020).

In Figure 5, we can observe that (1) the results in Figures 5B–D are better than one in Figure 5A, which shows that we can benefit from considering more source domains; (2) the result in Figure 5D is better than those in Figures 5B,C and which again validates the effectiveness of our model to adaptively evaluate the probability distribution and ensemble learning classifier.

**FIGURE 6:**
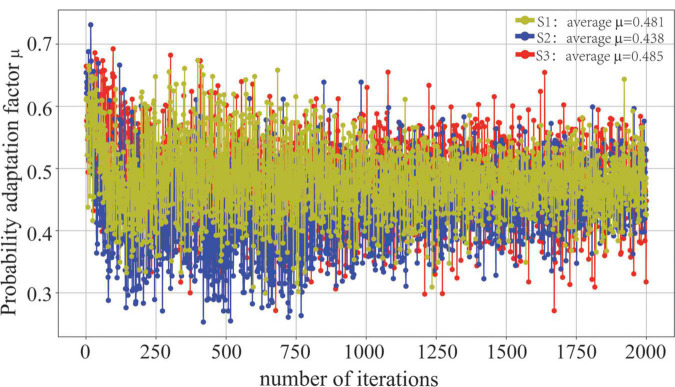
Visualization of the probability adaptation factor μ.

In addition, we evaluate the effectiveness of the probability adaptation factor μ, as shown in Figure 6. The average probability adaptation factors for S1, S2, and S3 are 0.481, 0.438, and 0.485, respectively. When μ→0, the difference in the global distribution between the source and target domains is large, and the marginal distribution adaptation is more important. When μ→1, it indicates that the difference in the local distribution between the source and target domains is higher, and the conditional distribution is more important. Because the S3 and target domains are homologous CT images, the global similarity (μ = 0.485) of the two images is higher, and it is necessary to focus on adapting their conditional distribution. Similarly, since S2 is a WSI image of the lung, it is quite different from the target domain in terms of the data generation mechanism and downstream tasks. Thus, it is necessary to focus on its global marginal probability distribution (μ = 0.438).

**FIGURE 7:**
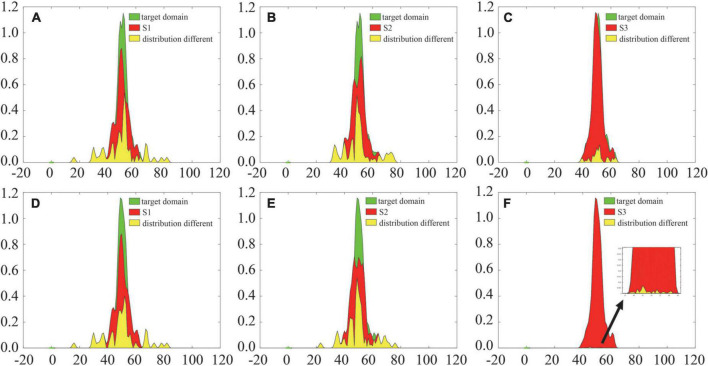
Comparison of probability density functions before and after probability distribution alignment. Panels **(A–C)** are the distribution differences before probability distribution alignment; panels **(D–F)** are the distribution differences after probability distribution alignment.

Furthermore, this manuscript uses kernel density estimation (KDE) (Dai et al., 2020), which is a nonparametric estimation method of the probability density function, to analyze the difference in the joint probability distribution between the source and target domains before and after alignment, as shown in Figure 7. The introduction of the adaptive probability distribution alignment strategy reduces the distribution difference between the source and target domains, and the change between S3 and the target domain is more obvious. The above results preliminarily verify the effectiveness of the adaptive alignment strategy, while the difference in S1 and S2 after alignment is still large. Thus, more effective difference measurement methods need to be introduced in future work.

## Conclusion

Aiming at the problem of preoperative diagnosis of GC and PGL under small samples, this manuscript proposes an ELM based on adaptation multiple spaces feature and L1-norm regularization (AMSF-L1ELM). From clinical practice, AMSF-L1ELM can learn the multiple source domain representation by performing dynamic distribution alignment between different source and target domains. We tested this on datasets from two centers, and the results demonstrate the superiority of AMSF-L1ELM over other state-of-the-art methods. However, there is still room for improvement in the performance of the proposed method, and more effective adaptation algorithms should be further studied in future work.

## Data availability statement

The original contributions presented in this study are included in the article/supplementary material, further inquiries can be directed to the corresponding author.

## Ethics statement

The studies involving human participants were reviewed and approved by Jiangmen Central Hospital Institutional Review Board. Written informed consent for participation was not required for this study in accordance with the national legislation and the institutional requirements.

## Author contributions

YL designed the research, collected the data, and wrote the manuscript. EC collected the data and supervised the study. Both authors contributed to the article and approved the submitted version.
